# Comparison of the Surgical Outcome between the Multiple Screw Fixation and Fixed Angle Devices for the Basicervical Femoral Neck Fractures

**DOI:** 10.3390/medicina60050680

**Published:** 2024-04-23

**Authors:** Jin-Woo Kim, Jung-Wee Park, Hyo-Jung Kim, Tae-Young Kim, Jun-Il Yoo, Young-Kyun Lee, Byung-Woong Jang

**Affiliations:** 1 Department of Orthopaedic Surgery, Nowon Eulji Medical Center, Seoul 01830, Republic of Korea; jinwu3911@hanmail.net (J.-W.K.); surgeon305@naver.com (H.-J.K.); 2 Department of Orthopaedic Surgery, Seoul National Bundang Hospital, Seongnam-si 13620, Republic of Korea; jwepark@gmail.com (J.-W.P.); ykleemd@gmail.com (Y.-K.L.); 3 Department of Orthopaedic Surgery, Konkuk University Hospital, Seoul 05030, Republic of Korea; syty-chan@hanmail.net; 4 Department of Orthopedic Surgery, Inha University Hospital, Inha University College of Medicine, Incheon 22332, Republic of Korea; furim@hanmail.net; 5 Department of Orthopaedic Surgery, Soonchunhyang University Seoul Hospital, Seoul 04401, Republic of Korea

**Keywords:** femoral neck fracture, multiple screw fixation, fixed angle device, surgical outcome

## Abstract

*Introduction*: Basicervical femoral neck fracture (FNF) is an uncommon type of femoral neck fracture and is associated with an increased risk of fixation failure due to its inherent instability. The purpose of this study was to compare the surgical parameters and reoperation rate between the use of a multiple cannulated screw (MCS) and fixed angle device (FAD) in treating basicervical FNFs. *Methods*: We retrospectively reviewed the records of 885 patients who underwent internal fixation between May 2004 and August 2019 to determine basicervical FNF with at least 12 months of follow-up. Among the identified 77 patients with basicervical FNF, 17 patients who underwent multiple cannulated screw (MCS) fixation and 36 patients who underwent fixed angle device (FAD) fixation were included. We compared the rates of fracture-site collapse and reoperations according to the fixation device. *Results*: Among the 53 patients with basicervical FNF, 13 patients (24.5%) sustained surgical complications (8 collapses of fracture site and 5 reoperations). The reoperation rate in the MCS group was significantly higher than that in the FAD group (23.5% vs. 2.8%, *p* = 0.016), without any significant difference in the collapse of the fracture site (11.8% vs. 16.7%, *p* = 0.642). *Conclusions*: Although basicervical FNF was rare among hip fractures, fracture site collapse was prevalent and prone to fixation failure. Surgeons should keep this in mind, and consider FAD for basicervical FNF.

## 1. Introduction

Femoral neck fracture (FNF) has been reported to account for approximately half of all hip fractures, and is one of the most serious fractures, as they are associated with high mortality and morbidity and reduced mobility in elderly patients [1]. Elderly patients with FNF require early surgical intervention to avoid serious medical complications such as pneumonia, pulmonary embolism, deep vein thrombosis, urinary tract infections, and pressure sores [2,3,4]. Closed reduction and internal fixation have been proposed as surgical options for non-displaced FNF [5].

Basicervical FNF is an uncommon type of femoral neck fractures that occurs at the junction of the base of the femoral neck with the intertrochanteric region [6] and represents an intermediate form between intracapsular and extracapsular fractures [7,8]. Biomechanical stability can be easily compromised even after internal fixation due to the mismatch of the cortical bone between the proximal and distal fragments. Few studies have shown that internal fixation for basicervical FNF increases the risk of fixation failure and reoperation [1,6,9,10,11,12,13,14,15,16,17,18].

Multiple cannulate screw (MCS) fixation has been most widely used to fix FNF in elderly patients [19,20]. Recently, few studies on internal fixation for FNF showed that a fixed angle device (FAD) had a better clinical outcome in treating mechanically unstable FNF, such as Pauwel type 3, compared with MCS [1,6,10]. However, only a few studies compared the surgical parameters and reoperation rate when treating basicervical FNF with MCS fixation and with FAD, including those using a cephalomedullary nail (CMN) [5,6,12,14,21]. 

Therefore, the purpose of this study was to compare the complication and reoperation between the use of MCS and FAD for basicervical FNF.

## 2. Materials and Methods

### 2.1. Study Population 

Between May 2004 and August 2019, we retrospectively reviewed the medical records of 885 FNFs aged 50 years or older treated at four tertiary referral hospitals.

**Figure 1:**
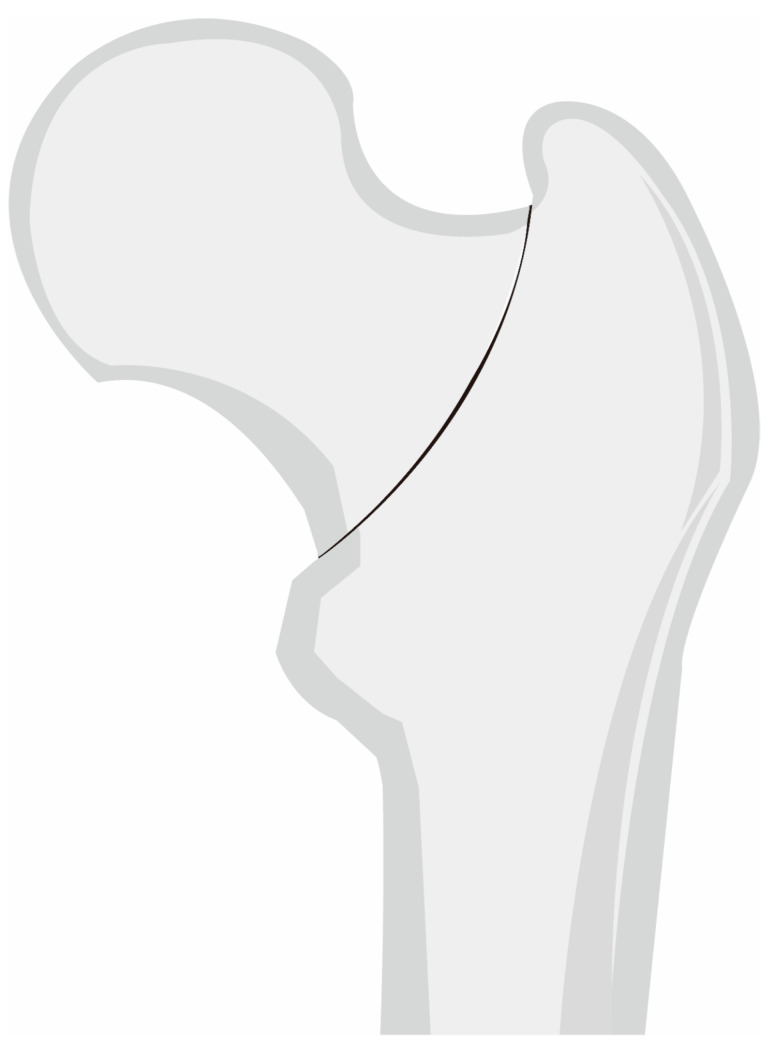
Schematic figure depicting a radiograph according to the definition of basicervical FNF in this study. FNF, femur neck fractures.

Basicervical FNF was identified by the method used by Watson et al. [1]. It was defined as the two-part fracture of the proximal femur that has its fracture line through the base of the femoral neck in conjunction with the intertrochanteric line, and exits between the lesser trochanter and the medial cortical breakage of the classic transcervical fracture [1] (Figure 1). Fractures in which the lesser trochanter was a separate fragment, or where the fracture line was distal to the lesser trochanter or outside the lateral cortex of the greater trochanter, were excluded. Additionally, concomitant fractures and periprosthetic fractures were excluded.

**Figure 2:**
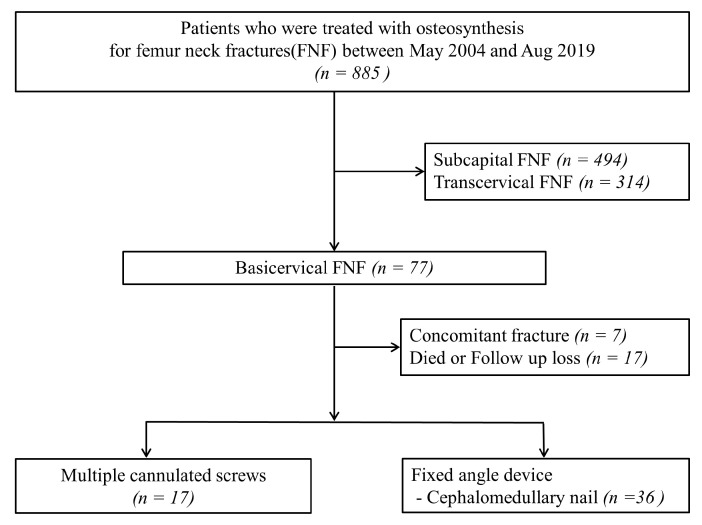
Flowchart to identify basicervical FNF patients. FNF, femur neck fractures.

Of the 885 FNF, 77 (8.7%) were classified as basicervical FNFs. Among them, patients who died within 1 year after surgery or were lost to follow-up (17 patients), and those with concomitant ipsilateral femur shaft fractures (7 patients) were excluded. Finally, of the remaining 53 patients with basicervical FNF included in the study, 36 patients were in the FAD group and 17 patients were in the MCS group (Figure 2). All included patients were evaluated with a hip anteroposterior, cross-table lateral radiographs, and hip 3D CTs. 

Demographic data including age, sex, body mass index (BMI), Charlson comorbidity index (CCI) [22], walking ability by Koval’s grade [23], Dorr type for cortical thickness of the proximal femur [24], and American society of anesthesiologists (ASA) score [25] were recorded preoperatively. All 36 cases that used FAD were treated with CMNs. There were no cases in which sliding hip screws (SHS) or Femoral Neck Systems (DePuy Synthes, Raynham, MA, USA) were used in this study.

### 2.2. Surgical Technique

All surgeries were performed by six orthopedic board-certified surgeons with a minimum of 5 years of experience in trauma surgery. The surgeons practicing in each respective centers were fellowship-trained in the same tertiary academic hospital.

Closed reduction by image intensifier was performed in all patients. After traction, the affected leg was abducted, adducted, and rotated to reduce fractures. An acceptable reduction was when the neck-shaft angle was reduced to within 5 degrees and the fracture displacement was less than 4 mm compared to the normal side.

In the MCS group, an incision on the mid-lateral aspect of the greater trochanter was made to place guide wires from the lateral cortex of the femur toward the femoral head via the femoral neck. Three guide wires were inserted in parallel reverse triangle configuration as near as possible to each respective cortex (inferior, posterosuperior, and anterosuperior) of the femoral neck. After confirming the trajectories were safely located inside the femoral neck with the appropriate tip–apex distance of the guide pins, the lengths of the guide pins were measured and the corresponding cannulated screws were inserted in the respective sites [26,27]. 

In the FAD group, CMNs were used in all cases. An incision was made along the proximal extension of the lateral side of the greater trochanter for the nail entry. A guide wire was placed in the center of the femoral canal through the greater trochanter tip. After proximal reaming through this site, the nail was inserted with the target device assembly attached in the proximal end. The guide pin for the proximal screw or blade was inserted into the center of the femoral head. Center to the inferior position in the anteroposterior view and the center position in the cross-table lateral view was deemed acceptable. A proximal screw or blade was inserted following the guide wire, and the distal locking screw was fixed to prevent the rotation of the nail [28].

After the respective surgery, an individualized rehabilitation protocol was prescribed with the emphasis on the early mobilization. Active range-of-motion exercise was indicated immediately after the surgery. From the day after the surgery, wheelchair use was encouraged. The patients were educated to start protective weight-bearing using assistive devices (a walker, crutches, or a cane) as possible 3~10 days after the operation [29]. Afterwards, the respective devices were changed by a physical therapist according to the respective conditions. For those patients who were unable to ambulate due to medical comorbidities, the use of wheelchairs and prevention of pressure sores were primarily indicated. The rehabilitation protocol was not different according to the implants used for fixation.

### 2.3. Outcome Variables

Operative data including the time to surgery, type of anesthesia, operation time, estimated blood loss during operation, and length of hospital stay were obtained by reviewing medical records.

The radiographic evaluation was performed by two independent observers who did not participate in the surgery. Radiographs immediately after surgery were used as a baseline for radiographic comparison. Routine follow-up was scheduled after 6 weeks, after 3,6,9, and 12 months, and every year thereafter. 

We compared the rates of fracture site collapse and reoperations according to the fixation device used between patients treated with MCS and CMN. 

The collapse of the fracture site was determined by comparing the immediate postoperative and the latest anteroposterior radiographs. A change in fracture position by more than 10 mm or in the screw position by more than 5%, and the backing of the screws by more than 20 mm, were defined as a collapse of the fracture site [30].

Reoperation was defined as any reoperation such as conversion to hip arthroplasty due to the severe collapse of the fracture site, osteonecrosis of femoral head, and fixation failure. For the diagnosis of osteonecrosis of the femoral head, anteroposterior radiograph of the hip was primarily evaluated. In the early stages, however, the necrotic border is not conspicuous in the radiograph. Therefore, the MRI or bone scintigraphy were used for more accurate detection, with a preference for the latter due to the metal artifact decreasing the sensitivity of the MRI. 

### 2.4. Statistical Analysis

Prior to conducting statistical tests, the distribution of continuous data were evaluated using the Shapiro–Wilk test to determine normality. Age, BMI, follow-up period, CCI, and ASA score followed normal distribution, and Student’s t-test was employed to compare means between the MCS and FAD groups. Time to surgery, operation time, estimated blood loss, and the length of hospital stay did not follow normal distribution, and the Mann–Whitney U test was used as a non-parametric alternative to assess statistical differences between the two groups.

The level of significance for all statistical tests was set at *p* < 0.05. This threshold was used to determine the presence of statistically significant differences between the two treatment groups. Categorical variables were presented as number (%) and continuous variables were presented as mean ± standard deviation. All statistical analyses were performed with the SPSS for Windows statistical package version 20.0 (SPSS, Chicago, IL, USA). 

### 2.5. Ethical Approval

The design and protocol were approved by each hospital’s Institutional Review Board, which waived the requirement for informed consent. This study was conducted in accordance with The Code of Ethics of the World Medical Association (Declaration of Helsinki). Approval for the study was obtained from the concerned Institutional Review Board (Nowon Eulji Medical Center 2019-10-015). The requirement for informed consent was waived by the Nowon Eulji Medical Center Helsinki committee because this is a retrospective analysis of deidentified data.

## 3. Results

The mean follow-up duration was 25.5 ± 14.0 months in the MCS group and 21.8 ± 7.7 in the FAD group (*p* = 0.213). The MCS patients (65.4 ± 12.0) were significantly younger than the FAD patients (79.6 ± 8.0) (*p* < 0.001). Among them, the number of patients aged 65 years or older was 8 in the MCS groups (47.1%, 8/17) and 34 in the FAD group (94.4%, 34/36). The CCI in the FAD group (0.5 ± 0.7) was higher than that in the MCS group (1.9 ± 1.6) (*p* < 0.001). The ASA score in the FAD group (1.6 ± 0.6) was also higher than that in the MCS group (2.2 ± 0.5) (*p* = 0.002) (Table 1). Operation time, estimated blood loss, and length of hospital stay were similar in both groups.

Eight patients sustained collapse of the fracture site: two in the MCS (11.8%) group, and six in the FAD group (16.7%) (*p* = 0.642). Four (23.5%) patients in the MCS group underwent reoperation due to two fracture site collapses and two osteonecrosis of femoral head (ONFH), whereas one (2.8%) of the six fracture site collapses required reoperation in the FAD group (*p* = 0.016) (Table 2).

## 4. Discussion

The proportion of basicervical FNF was 8.7% (77/885) of femoral neck fracture in this large multicenter study. Among the 53 basicervical FNF that were for followed-up at least 1 year, 15.1% (8/53) showed fracture site collapse regardless of the fixation device. Moreover, 9.4% (5/53) required reoperation.

Previous studies have also demonstrated that basicervical FNFs are relatively rare, accounting for only 1.8 to 11.6% of all hip fractures [7,8,31,32]. Our results (8.7%, 77/885) were compatible with these. 

Among the 53 basicervical FNF that were for followed-up at least 1 year, there was no difference of fracture site collapse between MCS and FAD (11.8% vs. 16.7%, *p*-value = 0.642). However, the reoperation rate in the MCS group was significantly higher than in the FAD group (23.5% vs. 2.8%, *p* = 0.016). Our results were comparable with previous studies (Table 3). Other clinical studies have reported good surgical outcomes for FAD in treatment of basicervical FNF. However, Watson et al. [1] reported a high reoperation rate (45.5%, 6/11), but this is a single-arm study and there is no comparison, so it is difficult to compare it directly. Chapman et al. [33] also reported a reoperation rate of 33.3% (2/6), but the study had a small number of patients.

**Figure 3:**
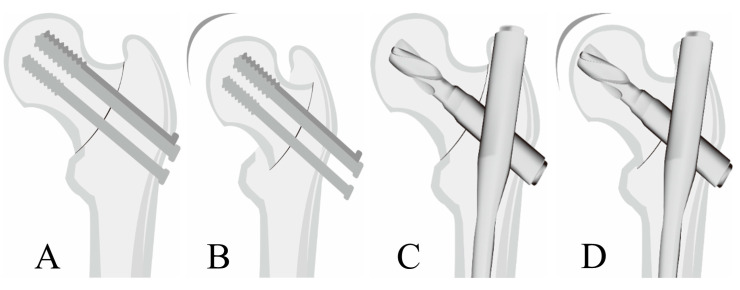
Schematic figure depicting a radiograph of MCS or FAD for basicervical FNF. (**A**) MCS for basicervical FNF. (**B**) Fracture site collapse after MCS. (**C**) CMN for basicervical FNF. (**D**) Fracture site collapse after CMN and no further collapse because of buttress against fracture site collapse. FNF, femur neck fractures; MCS, multiple screw fixation; FAD, Fixed angle device; CMN, CMN.

Biomechanical studies also support this by suggesting that FAD is better. In a biomechanical study, the CMN supported higher loads until failure and resulted in lesser displacement of the femoral head than those caused by cannulated screw fixation [7]. Figure 3 demonstrates that the CMN could be the buttress against fracture site collapse (Figure 3C,D) and prevent the further collapse of fracture site, compared with MCS for basicervical FNF (Figure 3A,B). 

It was notable that the FAD group showed shorter operation time and more blood loss compared to the MCS group. Among FAD, SHSs require wider incision to purchase the lateral wall to screw the plate to the lateral cortex. However, the CMN requires smaller incision that is designated by the target device attached to the proximal end of the nail. Therefore, the operation time and bleeding is typically less than the SHS [34,35]. Similar to SHS, MCS requires certain length of incision to approach the lateral cortex and require surgical accuracy to purchase near the cortical boundaries in reverse triangle configuration. Even the experienced surgeons sometimes need to revise the trajectory of the guide pins many times before the insertion of the cannulated screws. In contrast, the CMN requires only one guide pin placement each in the center of the femoral canal and the center of the femoral head through the femoral neck. Other than this, the fixed angle target device mandates the trajectory and this quickens the rest of the process.

Our results favoring FAD in basicervical FNF were comparable with that of previous studies. In the previous reports, reoperation rates of MCS for basicervical FNF were 16.1% (5/31) [5] and 42.9% (3/7) [6], while those of CMNs were 0% (0/13) [6], and dynamic hip screws were 11.1% (5/45) [21]. In a systematic review, the reoperation rate of cancellous screw fixation was 22.5% (9/40 patients), which was like the results of our study. The high failure rate of cancellous screw fixation was related to the lower fixation strength than observed in FAD [36,37]. Interestingly, the collapse of the fracture site tended to be more common in FAD group than in MCS group (16.7% vs. 11.8%, *p* = 0.642) even though the subsequent revision rate was significantly lower in the FAD group. This finding suggests that the FAD, or specifically CMN in this study, allows more collapse without the loss of stability in the fracture site compared to the MCS. In a biomechanical study, Blair et al. [36] showed that MCS had a significantly lower ultimate axial load to failure than FAD. In an unstable FNF model, fixation with CMNs were more durable than the MCS. The authors stated that the CMN allows the load to be transferred from the femur neck to the diaphysis, which is not possible in MCS [38]. In this study, patients in the MCS group were younger and had lower comorbidities and ASA scores, but the reoperation rate was significantly higher than that in the FAD group.

In the present study, osteonecrosis occurred in 11.8% (2/17) patients of the MCS group, while no osteonecrosis was reported in the FAD group. In a previous randomized study, avascular or reduced vascularity using bone scintigraphy was observed in 10.6% (5/47) patients in the four AO cancellous screw group [39]. In young patients, osteonecrosis occurred after femoral neck fractures in 23% of patients [40]. There were no previous reports comparing the rate of osteonecrosis after the use of MCS and CMNs. The proximal screw or blade in the nail system is targeted to the center or inferior part of the femoral head. However, in MCS, the screws are placed as far from each other as possible in the reverse triangular configuration. This might increase the possibility of placement of guide wire outside the femoral neck, invading the extracapsular arterial ring or epiphyseal arteries. This could be the potential cause of the subsequent osteonecrosis, but the number of patients were very limited, and further studies should be conducted for robust evidence.

This study had several limitations. Firstly, this is a retrospective study, and the number of patients was too small to determine risk factors for reoperation. The number of patients included in each group was small to conduct a subgroup analysis. However, considering basicervical FNF is a relatively rare condition, a study with a large number of patients could not be always feasible. We did not perform a sample size calculation, as this was a retrospective study. Additionally, a post hoc power analysis revealed that our study achieved a power of 74.3%, which is reasonably substantial given the rarity of the condition and the challenges associated with recruiting larger samples. Secondly, we were unable to analyze the bone mineral density (BMD), which could affect the overall study outcome as BMD assessment was available only in some patients. Thirdly, there might be a selection bias because older patients in the FAD group could be less active compared to the MCS group. However, there was no difference of walking ability between both groups. Fourthly, we could not conduct a cost analysis in this multicenter study because we could not evaluate an indirect cost such as utility for each condition. Fifthly, the time to surgery varied greatly, as it could be seen in the high standard deviation (7.8 days and 4.1 days) in both groups. This is due to the time consuming medical clearance for the underlying comorbidities in the elderly hip fracture patients. In South Korea, there are many tertiary hospitals where very specific exams, notably echocardiography, are required prior to the hip fracture surgery to evaluate the cardiac status. The hospital stays include the time to surgery because almost all hip fracture patients are admitted through the emergency department and start to be evaluated for medical clearance before the surgery.

## 5. Conclusions

Although basicervical FNFs are a rare type of FNF, fracture site collapse was prevalent, regardless of the fixation device. When choosing fixation devices for basicervical FNFs, the rate of reoperation is less in FAD compared to MCS. Further studies with larger cohorts are warranted to evaluate the surgical outcomes of basicervical FNFs

## Figures and Tables

**Table 1:** Comparison of demographic data according to fixation device.

Parameters	MCS (*n* = 17)	FAD (*n* = 36)	*p*-Value
Sex (male:female), patients	7:10	9:27	0.231
Age, years *	65.4 ± 12.0	79.6 ± 8.0	<0.001
BMI, kg/m^2^ *	21.8 ± 2.8	21.7 ± 3.6	0.894
FU period, months	25.5 ± 14.0	21.8 ± 7.7	0.213
Side (left:right), patients	11:6	21:15	0.658
CCI (Charlson Comorbidity index) *	0.5 ± 0.7	1.9 ± 1.6	<0.001
Walking ability, patients (%) §			0.122
Outdoor (Koval’s grade I–III)	14 (82.4%)	22 (61.1%)	
Housebound (Koval’s grade IV–VII)	3 (17.6%)	14 (38.9%)	
Dorr classification			0.045
Type A	3 (17.6%)	4 (11.1%)	
Type B	13 (76.5%)	18 (50.0%)	
Type C	1 (5.9%)	14 (38.9%)	
ASA score	1.6 ± 0.6	2.2 ± 0.5	0.002

* Expressed as number (%) for categorical variables and mean ± standard deviation for continuous parametric variables. ^§^ modified from Koval’s grade [14]. FNF, femur neck fractures; MCS, multiple screw fixation; FAD, Fixed angle device; *n*, numbers; FU, follow-up; BMI, body mass index; ASA, American Society of Anesthesiologists; CCI, Charlson’s Comorbidity Index.

**Table 2:** Comparison of surgery-related parameters between patients according to fixation device.

Parameters	MCS (*n* = 17)	FAD (*n* = 36)	*p*-Value
Type of anesthesia, patients (%)			0.570
Regional	12 (70.6%)	28 (77.8%)	
General	5 (29.4%)	8 (22.2%)	
Time to surgery, days *	1.0 (0.5–7.5)	3.0 (2.0–4.0)	0.507
Operation time, minutes *	50 (40–66)	49 (40–54)	0.066
Estimated blood loss, mL *	50 (40–100)	50 (50–100)	0.303
Length of hospital stays, days *	16 (6–25)	9.5 (8–16.25)	0.654
Complications			0.642
Fracture site collapse	2 (11.8%)	6 (16.7%)	
Osteonecrosis	2 (11.8%)	0	
Reoperation	4 (23.5%)	1 (2.8%)	0.016

* Expressed as number (%) for categorical variables and median (Q1–Q3) for continuous nonparametric variables. FNF, femur neck fractures; MCS, multiple screw fixation; FAD, Fixed angle device; *n*, numbers.

**Table 3:** Previous studies about surgical outcomes after MCS or FAD for basicervical FNF.

Study	No. of Patients	Type of Implants	Fixation Failure Rate	Reoperation Rate
Watson et al. (2016) [1]	11	CMN	54.5% (6/11)	45.5% (5/11)
Chapman et al. (2018) [33]	6	CMN	33.3% (2/6)	33.3% (2/6)
Tasylkan et al. (2015) [18]	25	CMN	0	0
Okano et al. (2017) [16]	14	CMN	0	0
Kweon et al. (2017) [31]	15	CMN (Gamma 3 nail)	0	0
Massoud (2010) [15]	13	Gamma nail or DHS or cancellous screw	0	0
Su et al. (2006) [17]	28	DHS	17.9% (5/28)	17.9% (5/28)
Chen et al. (2008) [9]	269	DHS	2.2% (6/269)	2.2% (6/269)
Kuokkanen (1991) [13]	6	DHS	0	0
Lee et al. (2018) [14]	69	DHS	17.2% (5/29)	17.2% (5/29)
		PFNA	2.5% (1/40)	2.5% (1/40)
Kim et al. (2020) [12]	106	DHS	12.8% (5/39)	2.6% (1/39)
		CMN	7.5% (5/67)	6.0% (4/67)
Enocson and Lapidus (2012) [11]	93	DHS	16.1% (15/93)	16.1% (15/93)
Saarenpää et al. (2002) [6]	30	CMN	0	0
		DHS	10% (1/10)	10% (1/10)
		CS	42.9% (3/7)	42.9% (3/7)
Nauth et al. (2017) [5]	76	DHS	Not mentioned	11.1% (5/45)
		CS		16.1% (5/31)
Sharma et al. (2018) [21]	90	CS	17.2% (5/29)	Not mentioned
		DHS	3.7% (1/27)	
		PFN	3.1% (1/32)	

CMN; CMN, PFNA; proximal femoral nail-antirotation, DHS; dynamic hip screw, CS; cannulated screw, PFN; proximal femoral nail.

## Data Availability

The datasets used and analyzed during the current study are available from the corresponding author on reasonable request.
